# Risk-adapted first-line therapy selection in advanced EGFR-mutant NSCLC: a practical clinical algorithm integrating tumor biology and patient factors

**DOI:** 10.1007/s12032-026-03338-y

**Published:** 2026-08-29

**Authors:** Ashish Sharma, Joecelyn Kirani Tan, Harendra Kumar, Hareesha Rishab Bharadwaj

**Affiliations:** 1 Department of Internal Medicine, Yale New Haven Hospital, New Haven, CT 06510 USA; 2 Faculty of Biology, Medicine and Health, University of Manchester, Manchester, M13 9PL UK; 3 Department of Internal Medicine, Mayo Clinic, Rochester, MN 55905 USA; 4 Department of Internal Medicine, Royal Stoke University Hospital, University Hospitals of North Midlands NHS Trust, Stoke-on-Trent, UK

**Keywords:** EGFR-mutant non-small cell lung cancer, Amivantamab-lazertinib, Osimertinib, MARIPOSA, FLAURA2, Risk-adapted treatment selection

## Abstract

Advanced *EGFR*-mutant non-small cell lung cancer (NSCLC), specifically tumors harboring classical activating mutations (exon 19 deletions and L858R substitutions, which are the exclusive focus of this review), is no longer a single-treatment landscape. The MARIPOSA and FLAURA2 phase 3 trials have established that combination regimens of amivantamab plus lazertinib and osimertinib plus platinum-pemetrexed, respectively, extend both progression-free and overall survival compared with osimertinib monotherapy. Each carries substantially greater toxicity, treatment complexity, and cost. No validated, prospective framework exists to guide first-line selection between combination and single-agent strategies. Decision-making is currently anchored to trial eligibility criteria and institutional norms rather than individualized risk stratification. This review synthesizes evidence from landmark trials, molecular biomarker analyses, and real-world cohort studies to propose a practical risk-adapted algorithm integrating tumor biology, molecular co-alterations, patient performance status, comorbidities, logistical feasibility, and treatment preference. Patients with biologically high-risk features, including liver metastases, baseline central nervous system (CNS) involvement, circulating tumor DNA (ctDNA) elevation, or heavy disease burden, may derive the greatest absolute benefit from combination therapy. *TP53* co-mutation warrants consideration but should be interpreted as a prognostic marker rather than a definitive treatment-selection criterion given conflicting predictive data across trials. The subcutaneous (SC) formulation of amivantamab, demonstrated in PALOMA-3 to reduce infusion-related reactions and administration time relative to the intravenous formulation, may improve the tolerability and logistical feasibility of MARIPOSA-based therapy, though prospective comparative evidence across formulations remains limited. Prospective biomarker-driven trials are needed to validate treatment selection; until that evidence matures, individualized shared decision-making guided by the proposed algorithm is the most defensible clinical approach.

## Introduction: the emerging treatment dilemma

*EGFR*-mutant non-small cell lung cancer (NSCLC) accounts for approximately 15% to 50% of nonsquamous lung adenocarcinomas, with marked prevalence variation by ethnicity and geography [1]. Classical (common) activating mutations, exon 19 deletions and the L858R substitution, together account for approximately 80% to 90% of *EGFR* alterations in this population and define the patient group for whom contemporary first-line strategies, including the MARIPOSA and FLAURA2 combinations, have been validated; the scope of this review is restricted to this group. The FLAURA trial established Osimertinib as a potent, selective, irreversible third-generation *EGFR* tyrosine kinase inhibitor (TKI) with central nervous system (CNS) penetrance as the preferred first-line therapy, demonstrating a median progression-free survival (PFS) of 18.9 months and a final overall survival (OS) of 38.6 months, substantially exceeding first-generation comparators [2, 3]. For several years, the primary clinical question was logistical: whether osimertinib was accessible, rather than whether it was optimal.

That relative simplicity has now been disrupted by two pivotal phase 3 trials. In MARIPOSA, the chemotherapy-free combination of amivantamab, a fully human bispecific antibody targeting *EGFR* and *MET* with immune cell-directing activity plus lazertinib, a brain-penetrant third-generation *EGFR* TKI, produced a median PFS of 23.7 months and a statistically significant OS advantage over osimertinib (hazard ratio (HR) 0.75; 95% confidence interval (CI) 0.61–0.92; *P* = 0.005), with a 3-year OS of 60% versus 51%, and a median OS for the combination arm that had not been reached at final analysis [4, 5]. In FLAURA2, adding platinum-pemetrexed to osimertinib achieved a median PFS of 25.5 months and a final OS of 47.5 versus 37.6 months (HR 0.77; 95% CI 0.61–0.96; *P* = 0.02) [6, 7]. Both combinations have received Food and Drug Administration (FDA) approval and carry the highest recommendations in the American Society of Clinical Oncology (ASCO) Living Guideline 2025.1 and National Comprehensive Cancer Network (NCCN) guidelines [8, 9].

Yet this therapeutic expansion creates an unavoidable clinical dilemma. Combination regimens impose substantially greater toxicity, more frequent clinical encounters, and higher cost. Final OS analysis data report grade ≥ 3 adverse events in 70% of FLAURA2 combination patients and 80% of MARIPOSA combination patients [5, 7]. Not every patient with *EGFR*-mutant NSCLC needs or can safely tolerate this degree of treatment intensification. Both pivotal trials enrolled patients with Eastern Cooperative Oncology Group (ECOG) performance status 0 or 1, with median ages in the early-to-mid 60s, leaving clinicians without a validated selection framework for the broader and more heterogeneous real-world patient population.

The critical clinical question has therefore shifted: it is no longer whether combination therapy works, but who should receive it. This review synthesizes the most current evidence and proposes a risk-adapted treatment algorithm to guide individualized first-line decision-making.

Scope and objectives. This review focuses exclusively on advanced NSCLC harboring classical activating *EGFR* mutations, defined as exon 19 deletions and the L858R substitution. Uncommon variants, including exon 20 insertions, de novo T790M, compound mutations, and atypical alterations in other exons, are biologically and therapeutically distinct, are not addressed by the MARIPOSA or FLAURA2 evidence base, and fall outside the scope of this work. Our objectives are threefold: (i) to synthesize landmark trial data, biomarker analyses, and real-world cohort findings relevant to first-line decision-making in this defined population; (ii) to propose a transparent, risk-adapted framework integrating tumor biology, molecular co-alterations, patient fitness, and logistical feasibility; and (iii) to articulate the limitations of current evidence and the prospective validation steps required before the framework can be regarded as practice-defining.

## Evidence overview: combination versus single-agent therapy

Three regimens now carry regulatory approval or high-grade guideline endorsement for first-line treatment of *EGFR*-mutant (exon 19 deletion or L858R) advanced NSCLC: osimertinib monotherapy, osimertinib plus platinum-pemetrexed (FLAURA2), and amivantamab-lazertinib (MARIPOSA). No direct, head-to-head comparison of all three exists.

Osimertinib monotherapy, the former universal standard established by FLAURA, delivers durable responses with a well-characterized and manageable adverse event profile. Grade ≥ 3 events occurred in 42% of patients in the primary analysis, and a final median OS of 38.6 months represents a meaningful benchmark [2, 3]. Real-world data from a multicenter prospective Japanese registry of 583 patients confirmed median PFS of 20.0 months and OS of 41.0 months under routine clinical practice conditions, with grade ≥ 3 adverse events in 23% of patients [10].

The FLAURA2 trial enrolled 557 patients with *EGFR*-mutated advanced NSCLC (ECOG 0–1) and randomly assigned them to osimertinib alone or osimertinib with four cycles of intravenous platinum-pemetrexed induction followed by continuous pemetrexed maintenance [6]. Investigator-assessed PFS improved significantly (median 25.5 versus 16.7 months; HR 0.62; *P* < 0.001). Final OS data confirmed a statistically significant survival benefit (47.5 versus 37.6 months; HR 0.77; *P* = 0.02), consistent across prognostic subgroups [7]. Grade ≥ 3 adverse events in the final OS analysis were observed in 70% of combination patients versus 34% on monotherapy, an increase driven primarily by hematologic toxicity [7]. Only 77% of patients completed all four induction chemotherapy cycles; any-cause treatment discontinuation was markedly higher in the combination arm (48% versus 6%), and osimertinib dose interruptions occurred in 43% of combination patients [6]. Patient-reported outcomes from FLAURA2 demonstrated a transient decline in global health status during the induction phase, with recovery thereafter during pemetrexed maintenance, a trajectory that merits explicit discussion with patients before treatment initiation [11].

MARIPOSA enrolled 1,074 patients randomized 2:2:1 to amivantamab-lazertinib, osimertinib, or lazertinib monotherapy [4]. The combination demonstrated a median PFS of 23.7 months versus 16.6 months with osimertinib (HR 0.70; 95% CI 0.58–0.85; *P* < 0.001). At final OS analysis (median follow-up 37.8 months), amivantamab–lazertinib produced significantly longer survival (HR 0.75; *P* = 0.005), with 3-year OS of 60% versus 51% and a median OS that remained not reached [5]. Time to symptomatic progression was prolonged by more than 14 months compared with osimertinib (43.6 versus 29.3 months; HR 0.69; *P* < 0.001) [5]. Grade ≥ 3 adverse events were observed in 80% of patients at final analysis, predominantly comprising *EGFR*- and *MET*-mediated dermatologic events, infusion-related reactions (IRRs), and venous thromboembolic events (VTE) [5].

The approval and adoption of subcutaneous (SC) amivantamab administration substantially changes the risk-benefit profile of MARIPOSA-based therapy. The phase 3 PALOMA-3 trial demonstrated that SC amivantamab was non-inferior to intravenous (IV) administration in pharmacokinetic exposure, with a superior OS outcome (HR 0.62; *P* = 0.02) and a dramatic reduction in IRRs from 66% with IV to 13% with SC delivery [12]. Administration time decreased from approximately 5 h (IV) to under 5 min (SC), with substantially improved patient satisfaction and reduced healthcare resource utilization [13]. SC amivantamab is recognized as a preferred mode of delivery in current NCCN guidance and may be offered as the initial formulation when MARIPOSA-based therapy is selected, with the choice of route individualized to patient circumstances and infrastructure [9].

Table 1 provides a corrected, comprehensive comparative summary across efficacy, tolerability, CNS outcomes, and resistance data for all three first-line strategies.

**Table 1:** Comparative summary of first-line treatment approaches in EGFR-mutant advanced NSCLC

Parameter	Osimertinib Monotherapy (FLAURA)	Osi + Platinum–Pemetrexed (FLAURA2)	Amivantamab + Lazertinib (MARIPOSA)
Median PFS (months)	18.9	25.5	23.7
Median OS (months)	38.6	47.5 vs. 37.6	NR (vs. 42.9-NR; 95% CI)
3-year OS rate	N/A	57% vs. 46%	60% vs. 51%
OS HR (vs. osi mono)	Reference	0.77 (*P* = 0.02)	0.75 (*P* = 0.005)
Grade ≥ 3 AEs (final analysis)	42%†	70% vs. 34%†	80%‡
Route of administration	Oral daily	Oral + IV cycles → oral maintenance	Oral + SC amivantamab (preferred) or IV
Key toxicities	Rash, diarrhea, ILD (rare)	Cytopenias, nausea, fatigue, ILD	Rash, paronychia, VTE (40%), IRR (SC: 13%)
Treatment discontinuation	6% (any-cause)	48% (combination arm, any-cause)	Any-cause: 62% vs. 72% with osimertinib (at median 37.8-month follow-up; 38% vs. 28% remained on assigned therapy); TRAE leading to discontinuation of all agents: 10% vs. 3% [4, 5]
FLAURA2 CNS outcomes (median intracranial PFS)	13.8 months [14]	24.9 months (HR 0.56; FLAURA2)	N/A
MARIPOSA CNS outcomes (3-yr intracranial CNS-PFS)	18% [15]	N/A	38% (MARIPOSA)
Post-progression resistance	*MET* amp 13%, C797S 8%	ctDNA analysis (osi + CT vs. osi mono, *n* = 53 vs. 73): MET amplification 9% vs. 14%; EGFR C797S 4% vs. 12%; no novel resistance mechanisms specific to combination arm [16]	*MET* amp 3% (*P* = 0.002), C797S 1% (*P* = 0.01) vs. osi

AE = adverse event; CNS = central nervous system; HR = hazard ratio; ILD = interstitial lung disease; IRR = infusion-related reaction; NR = not reached; osi = osimertinib; SC = subcutaneous; VTE = venous thromboembolism.

†Grade ≥ 3 AE rates from final OS analyses (FLAURA2: Janné 2026; FLAURA monotherapy primary: Soria 2018). Primary analysis FLAURA2 rates were 64% vs. 27%.

‡MARIPOSA final analysis (Yang 2025); primary analysis (Cho 2024) reported 75%. CNS outcomes are trial-specific and not directly comparable across trials. They differ in end point definition, follow-up duration, and patient population. FLAURA2 CNS-PFS data from: Janné PA et al. J Clin Oncol. 2024;42(1):73–84 [Ref 14].

MARIPOSA 3-year intracranial CNS-PFS from: Gadgeel SM et al. IASLC WCLC 2024 [Ref 15]. Treatment discontinuation metrics are not uniformly defined across trials and reflect different follow-up durations. Any-cause discontinuation includes disease progression and is influenced by length of follow-up; treatment-related adverse event (TRAE) discontinuation captures discontinuation specifically attributable to toxicity and is more directly comparable across trials. Where available, both metrics are reported.

## Tumor biology and risk stratification

*EGFR*-mutant NSCLC is biologically heterogeneous. Outcomes on single-agent osimertinib vary substantially depending on the tumor’s molecular context and metastatic distribution. Several baseline features, anatomic and genomic, are consistently associated with inferior survival on monotherapy and may identify patients who could derive disproportionate benefit from treatment intensification. It is essential to note that most of this evidence derives from exploratory subgroup analyses and retrospective cohort data; no prospective biomarker-selection trial has yet validated any single variable as a definitive predictor of differential benefit.

### High-risk clinical and anatomic features

The site of metastatic disease carries independent prognostic significance in *EGFR*-mutant NSCLC. In a large US real-world cohort of 1,323 patients initiating first-line osimertinib monotherapy, median real-world OS was markedly lower in patients with liver metastases (19.3 months) or ECOG performance status ≥ 2 (18.1 months) compared with the overall population (28.6 months) [17]. In the MARIPOSA secondary biomarker analysis, the presence of liver metastases at baseline was associated with a numerically greater relative PFS benefit from amivantamab–lazertinib versus osimertinib, supporting the plausibility that visceral, high-burden disease responds preferentially to multi-pathway suppression [18]. Subgroup OS analyses from FLAURA2 also showed hazard ratios consistently favoring the combination in patients with bone and liver metastases [7].

Baseline CNS metastases represent another high-risk presentation requiring careful treatment consideration. FLAURA2 CNS analyses demonstrated that osimertinib plus chemotherapy produced a median intracranial PFS of 24.9 months compared with 13.8 months for monotherapy (HR 0.56), and intracranial objective response was improved regardless of the number of brain lesions [14]. In MARIPOSA, the 3-year intracranial CNS-PFS was 38% for amivantamab-lazertinib versus 18% for osimertinib, a clinically substantial difference [15]. It is important to note that these CNS end points derive from different analyses with different definitions and are not directly comparable across trials. Nonetheless, both combination strategies offer enhanced intracranial disease control relative to monotherapy, and in patients with active or multiple brain metastases, combination therapy merits strong consideration.

High overall disease burden at presentation, manifesting as symptomatic, rapidly progressive, or organ-threatening disease, is operationally relevant even where formal prognostic validation is lacking. In this context, the cytoreductive capacity of induction chemotherapy in the FLAURA2 framework may offer an earlier and deeper response that translates to meaningful clinical benefit.

### Molecular co-alterations: TP53 status and circulating tumor DNA

*TP53* is the most frequently co-altered tumor suppressor gene in *EGFR*-mutant NSCLC, identified in approximately 50% to 65% of tumors by comprehensive genomic sequencing [17]. Across multiple retrospective analyses, *TP53* co-mutation is consistently prognostic: patients with *TP53* co-alterations experience shorter TKI treatment duration and inferior OS with osimertinib monotherapy [19–21]. In one multicenter Brazilian cohort, *TP53* co-mutation was associated with a median time-to-treatment discontinuation of 18.7 months versus not reached in *TP53* wild-type patients, and a median OS of 30.4 versus 53.7 months [22].

However, the predictive value of *TP53* co-mutation, that is, whether it identifies patients who differentially benefit from combination over monotherapy, remains uncertain and must be interpreted with explicit caution. In the MARIPOSA secondary biomarker analysis, patients with *TP53* co-alterations showed a more pronounced absolute PFS benefit with amivantamab-lazertinib (HR 0.65; *P* = 0.003) compared with *TP53* wild-type patients (HR 0.75; *P* = 0.114), suggesting a possible predictive interaction [18]. In contrast, the FLAURA2 final OS analysis found no differential OS benefit by *TP53* status HRs consistently favored combination therapy in both *TP53*-mutated and *TP53* wild-type subgroups [7]. A prospective cohort study in patients with concurrent *EGFR* and *TP53* co-mutations similarly demonstrated PFS improvement with osimertinib plus chemotherapy versus monotherapy in this molecularly selected group [23]. The most direct prospective evidence to date comes from the phase 3 TOP trial (NCT06120140), presented at ELCC 2026, which randomized 294 patients with treatment-naive advanced non-squamous NSCLC harboring sensitizing *EGFR* mutations (exon 19 deletion or L858R) and concurrent *TP53* mutations to osimertinib plus carboplatin-pemetrexed (*n* = 146) or osimertinib monotherapy (*n* = 148) [24]. Investigator-assessed median PFS was 34.0 months with the combination versus 15.8 months with monotherapy (HR 0.44; P less than 0.001), with consistent benefit across prespecified subgroups including mutation subtype, brain metastasis status, and performance status [24]. Objective response rate (82.9% vs. 71.6%) and median duration of response (32.7 vs. 15.3 months) also favored the combination. Interim overall survival data, at 30.6% maturity, showed a favorable trend (median OS 48.4 vs. 36.5 months; HR 0.57), with mature OS data awaited. Grade 3 or higher treatment-related adverse events were substantially higher with combination therapy (62.4% vs. 14.9%), driven predominantly by hematologic toxicity, consistent with the FLAURA2 experience.

Several caveats are essential when applying these findings clinically. Both the MARIPOSA and FLAURA2 *TP53* analyses were exploratory, unplanned, and not powered for formal interaction testing. The MARIPOSA and FLAURA2 results are contradictory with respect to predictive directionality, and neither can be regarded as practice-defining in isolation. *TP53* mutation heterogeneity is clinically significant: mutations in the DNA-binding domain (particularly exon 5) appear to carry a more profound impact on osimertinib outcomes than mutations in other functional domains, though exon-level predictive stratification has not been prospectively validated [25]. Co-occurring alterations such as *PIK3CA* further complicate interpretation; in one US multi-institutional real-world cohort, *TP53* and *PIK3CA* co-alteration but not *TP53* alone conferred significantly worse OS [26].

In clinical practice, *TP53* co-mutation status should currently be regarded as a prognostic marker that supports heightened therapeutic vigilance rather than a definitive treatment-selection criterion. Clinicians should communicate this uncertainty clearly to patients. The phase 3 TOP trial discussed above (NCT06120140), prospectively evaluating osimertinib plus carboplatin-pemetrexed versus monotherapy specifically in *EGFR*-*TP53* co-mutated patients, has provided the first prospective, biomarker-selected evidence that combination therapy doubles PFS in this high-risk subgroup [24]. The TOP findings strengthen the rationale for treatment intensification in *TP53* co-mutated patients but should be interpreted with the caveats that the chemotherapy partner was carboplatin-pemetrexed (not amivantamab-lazertinib), final OS data remain immature, and the toxicity cost of combination therapy is substantial. Until OS data mature, *TP53* co-mutation should be regarded as a reasonable consideration favoring combination therapy in fit patients, particularly when paired with additional high-risk anatomic features.

Circulating tumor DNA (ctDNA) burden at baseline is an additional emerging risk marker. Detectable ctDNA at diagnosis correlates with higher systemic tumor dissemination and shorter osimertinib benefit duration in multiple retrospective analyses. In the MARIPOSA secondary analysis, patients with detectable baseline ctDNA showed a trend toward greater relative benefit from amivantamab-lazertinib [18]. While ctDNA-guided prospective treatment selection is not yet validated, quantitative ctDNA profiling represents a biologically credible axis for future biomarker-adaptive trial design.

### EGFR mutation subtype and additional genomic co-alterations

L858R mutation subtype has been variably associated with shorter osimertinib benefit duration compared with exon 19 deletions in some retrospective analyses, and the FLAURA2 final OS analysis suggested a numerically greater benefit from combination therapy in L858R patients [7]. However, MARIPOSA demonstrated statistically consistent benefit for amivantamab-lazertinib regardless of mutation subtype [4, 5]. Given these inconsistencies, L858R status should be regarded as a hypothesis-generating and exploratory factor supporting consideration of intensification not an established predictive biomarker for clinical decision-making. Co-occurring tumor suppressor alterations beyond *TP53*, including CDKN2A deletion, RB1 mutation, and concurrent multi-pathway suppressor co-alterations, have been associated with cumulative adverse prognostic impact in early analyses and warrant systematic evaluation in prospective biomarker-enriched studies [27].

## Patient-centered and practical considerations

### Toxicity profiles and prophylactic management strategies

Each combination regimen carries a distinct toxicity signature, and proactive prophylactic management is now an integral part of both treatment strategies. The practical impact of adverse events extends beyond their Common Terminology Criteria grade: even predominantly low-grade toxicities can impose significant quality-of-life impairment when sustained continuously over a multi-year treatment course.

For FLAURA2, dominant toxicity concerns arise during the chemotherapy induction phase (cycles 1 to 4), when hematologic events anemia, neutropenia, and thrombocytopenia are most pronounced. Standard antiemetic prophylaxis and nutritional support address gastrointestinal events. These toxicities generally attenuate after induction, but the 43% rate of osimertinib dose interruptions during induction is clinically significant given that sustained TKI exposure is pharmacologically critical [6]. Final analysis data report grade ≥ 3 events in 70% of combination patients [7]. Patient-reported outcome data from FLAURA2 show a temporary decline in global health status during induction, with recovery during maintenance; patients should be counseled about this anticipated trajectory before starting treatment [11].

For MARIPOSA-based therapy, dermatologic toxicity is the primary ongoing concern. Rash, paronychia, and periungual inflammation affect the majority of patients and are the most common drivers of dose modification and impaired quality of life. The COCOON trial evaluated a structured prophylactic dermatologic management protocol consisting of oral doxycycline or minocycline 100 mg twice daily for weeks 1 through 12, clindamycin 1% lotion applied to the scalp daily from weeks 13 to 52, chlorhexidine 4% applied to the nails daily, and a ceramide-based moisturiser applied to the face and body daily throughout treatment. Enhanced prophylaxis with the COCOON regimen reduced the incidence of grade ≥ 2 dermatologic adverse events from 75% in the standard care arm to 42% in the enhanced care arm (odds ratio 0.24; *P* < 0.0001) and also reduced patient-reported dermatologic symptom burden [28, 29]. The COCOON regimen is now considered standard of care and should be initiated simultaneously with amivantamab-lazertinib therapy.

IRRs, previously a major barrier to amivantamab tolerability, have been dramatically reduced with the SC formulation: IRRs fell from 66% with IV to 13% with SC amivantamab in PALOMA-3 [12]. For patients who receive IV amivantamab, the phase 2 SKIPPirr study (NCT05663866) prospectively evaluated four prophylactic strategies and identified an 8-mg oral dexamethasone regimen (twice daily on the two days preceding the first infusion and once 1 h before infusion on cycle 1 day 1) as the only approach to meet protocol criteria; this regimen reduced first-infusion IRR incidence from approximately 67% with standard premedication to roughly 22%, a threefold reduction, with no SKIPPirr participants discontinuing amivantamab because of IRRs [30]. This regimen is reflected in the current Rybrevant prescribing information and should be the default IRR prophylaxis whenever IV amivantamab is used [31].

VTE represents an independently important and underappreciated complication of amivantamab-lazertinib therapy. In the MARIPOSA primary analysis, VTE occurred in 37% of patients in the combination arm versus 9% with osimertinib; at final analysis, these rates were 40% versus 11%, with 62% of VTE events occurring within the first 4 months of treatment [4, 5]. Prophylactic anticoagulation with a low-molecular-weight heparin or direct oral anticoagulant during the first four months is now endorsed in clinical guidelines and should be discussed with all patients prior to initiating amivantamab [9, 32]. Prospective evidence supporting prophylactic anticoagulation comes from both PALOMA-2 and PALOMA-3. In PALOMA-2 (NCT05498428), VTE incidence with first-line SC amivantamab plus lazertinib was 18% in Cohort 1, in which prophylactic anticoagulation for the first four months was recommended (administered in 71% of patients), and 7% in Cohort 6, in which the same prophylaxis was mandated (administered in 100% of patients), with no dose reductions or discontinuations attributable to VTE and a 2% rate of grade 3 or higher VTE in the prophylaxis-treated population [33]. In PALOMA-3 (NCT05388669), 81% of patients received prophylactic anticoagulation; VTE rates were 10% in patients receiving prophylaxis compared with 21% in those not on prophylaxis, with low rates of clinically important bleeding across groups, and VTE rates were lower with SC than IV amivantamab (9% vs. 14%) [12]. Together, these data demonstrate that prophylactic anticoagulation can be safely implemented and approximately halves the VTE incidence with amivantamab-lazertinib, supporting its routine use in patients without contraindications. Patients with prior VTE, active malignancy-related hypercoagulability, or prolonged immobility represent the highest-risk subgroup for whom prophylaxis is particularly critical. Integrating these supportive care advances into routine practice is the explicit aim of the ongoing phase 2b COPERNICUS trial (NCT06667076), an open-label study currently enrolling participants across 123 US and 50 EMEA sites that pairs SC amivantamab plus lazertinib (first-line cohort) or SC amivantamab plus chemotherapy (second-line cohort) with structured, protocol-mandated prophylaxis for VTE and for dermatologic adverse events; the study uses pragmatic eligibility criteria to enroll a more clinically representative population than the MARIPOSA and MARIPOSA-2 registrational trials and will report on the integrated benefit-risk profile of toxicity-mitigated SC amivantamab regimens [34].

### Treatment logistics and access

Logistical considerations are central, not peripheral to treatment tolerability and adherence. The FLAURA2 regimen requires regular intravenous infusion visits during four cycles of induction and then ongoing pemetrexed maintenance infusions every three weeks indefinitely. This demands consistent access to infusion infrastructure, reliable transportation, and caregiver availability.

SC amivantamab fundamentally changes the logistical profile of MARIPOSA-based therapy. The transition from a 5-hour IV infusion to a subcutaneous injection requiring fewer than 5 min reduces clinic time commitment dramatically and substantially improves scheduling flexibility and patient-reported convenience [13]. SC amivantamab uses a different weight-based dosing schedule compared with the IV formulation, and the FDA-approved SC formulation can be substituted for IV per NCCN guidelines [9, 31]. For patients who would face genuine barriers to extended infusion access, SC amivantamab may transform MARIPOSA-based therapy from impractical to feasible.

Cost and insurance coverage are real-world implementation factors that clinical guidelines cannot prescribe, but clinicians cannot ignore. The incremental cost of combination regimens, particularly bispecific antibody therapy over oral TKI monotherapy, is substantial, with annual treatment costs for amivantamab-based therapy exceeding $200,000 USD in many health systems. In patients where out-of-pocket exposure is a meaningful adherence risk or where insurance prior-authorization delays are anticipated, these barriers must be incorporated into the shared decision-making discussion and addressed proactively through financial navigation resources.

### Shared decision-making framework

No algorithm substitutes for a structured, individualized conversation. The following practical checklist is designed to anchor first-line shared decision-making:

1. Is the patient’s ECOG performance status 0–1 and medically fit for intensified treatment?
2. Does the tumor harbor high-risk features: liver metastases, baseline CNS disease, *TP53* co-mutation (with acknowledgment of predictive uncertainty), elevated ctDNA, or high disease burden?
3. Does the patient understand and accept a higher toxicity burden including VTE risk and dermatologic adverse events, in exchange for a potential OS benefit?
4. Is infusion center or SC injection access feasible given the patient’s geography, transportation, and insurance?
5. Are there comorbidities, such as cardiac disease, prior VTE, renal impairment, frailty, or polypharmacy, that selectively elevate the risk of specific combination regimens?

When most of these questions yield affirmative, favorable answers and no contraindication is identified, combination therapy is appropriate. When one or more critical factors, particularly fitness, strong preference for lower toxicity, cost barriers, or logistical constraints, preclude intensification, single-agent osimertinib remains fully appropriate and guideline-endorsed.

## Special populations: geriatric and comorbid patients

The populations most likely to require individualized deintensification, elderly patients, those with significant comorbidities, and those with limited functional reserve are precisely those least well-represented in MARIPOSA and FLAURA2 trial populations. Both trials enrolled patients with ECOG 0 or 1, stringent organ function criteria, and median ages in the early-to-mid 60s. Real-world *EGFR*-mutant NSCLC patients are considerably more heterogeneous: in US real-world cohorts, the median age is approximately 70 years, and approximately one in five patients presents with ECOG ≥ 2.14.

For patients 75 years of age and older, the HOT2002 retrospective multicenter study (*n* = 132) demonstrated that first-line osimertinib monotherapy produced a median PFS of 19.4 months and a median OS that was not reached at analysis, outcomes closely approximating those in younger trial populations [35]. A second real-world study in this age group, OSI-FACT-EP (*n* = 203, age ≥ 75), reported a median PFS of 16.9 months and a median OS of 30 months under standard conditions, with 28.6% of patients discontinuing treatment due to adverse events [36]. For patients with poor performance status (PS 2–4), the OPEN/TORG2040 prospective phase 2 trial demonstrated that osimertinib monotherapy produced an objective response rate of 63.3%, median PFS of 8.0 months, and median OS of 25.4 months in this challenging group, though with a notable 20% rate of interstitial lung disease [37]. Taken together, these data reinforce that osimertinib monotherapy is an active and clinically meaningful option across a wide range of elderly and functionally compromised patients.

The anticipated tolerability of combination regimens in patients over 75 years is substantially inferior to that observed in trial populations. The 70% grade ≥ 3 toxicity rate in FLAURA2 final analysis and 80% in MARIPOSA final analysis are expected to be even higher in patients with reduced bone marrow reserve, baseline renal impairment, heightened fatigue sensitivity, and lower chemotherapy metabolic capacity. Although no elderly-focused combination therapy trial has been conducted, extrapolation from these data strongly supports monotherapy as the appropriate default in this age group, absent a compelling biological reason to escalate.

Frailty, distinct from chronological age and not fully captured by ECOG performance status, is an operationally essential concept. Validated screening tools such as the G8 questionnaire (which scores across nutritional status, mobility, neuropsychological profile, and functional decline) or the Clinical Frailty Scale can identify patients for whom treatment intensification poses disproportionate risk. ASCO and SIOG guidelines recommend formal geriatric assessment in older patients with cancer [8]. Frail patients who tolerate oral TKI therapy may still derive meaningful benefit from osimertinib without the physiological burden of combination strategies.

Polypharmacy and drug-drug interactions deserve specific attention in this population. VTE prophylactic anticoagulation required for amivantamab-based therapy interacts with the patient’s bleeding risk, concurrent cardiac and renal medications, and fall risk. Cytochrome P450 interactions with lazertinib are relevant in patients on complex cardiac, neurological, or anticoagulant regimens. A formal pharmacist review prior to combination initiation is strongly recommended for any patient over 70 years or on four or more concurrent medications.

## Proposed practical treatment algorithm

**Fig. 1:**
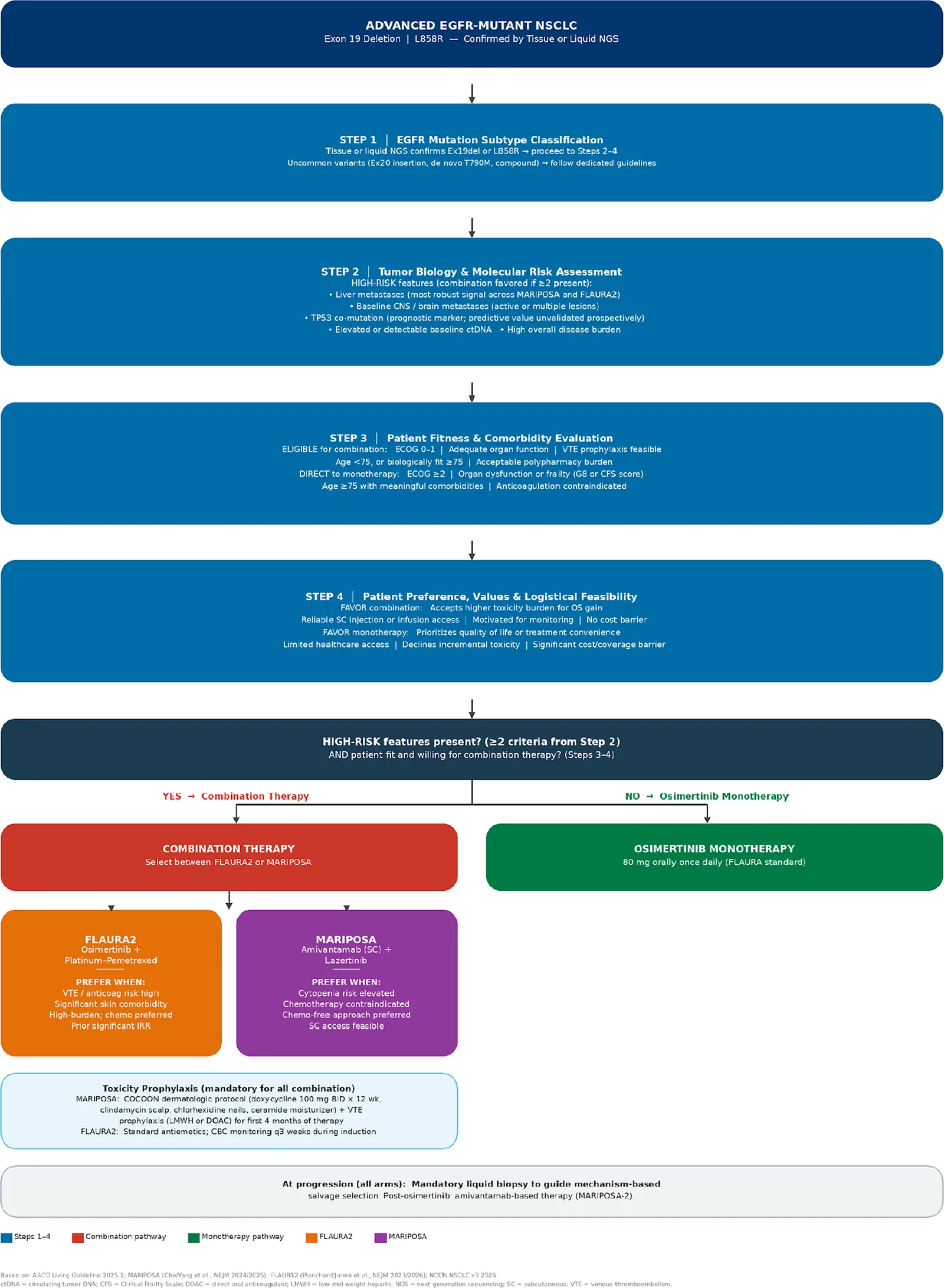
Proposed risk-adapted first-line treatment algorithm for advanced EGFR-mutant NSCLC

Integrating the evidence reviewed above, we propose a four-step risk-adapted framework for first-line treatment selection in advanced *EGFR*-mutant NSCLC (Fig. 1; Table 2). This algorithm is intended to guide individualized decision-making rather than prescribe a fixed approach.

Step 1: *EGFR* mutation confirmation and subtype classification. Confirm the specific actionable *EGFR* alteration (exon 19 deletion versus L858R versus uncommon variant) using tissue or liquid next-generation sequencing. Uncommon *EGFR* variants, including exon 20 insertions, de novo T790M, compound mutations, and atypical exons, may not share equivalent benefit from the regimens discussed here and should be managed per dedicated guideline recommendations.

Step 2: Tumor biology and molecular risk assessment. Evaluate for high-risk features: liver metastases (the most robust high-risk signal supported across multiple cohorts), baseline measurable CNS disease, *TP53* co-mutation (interpreted as a prognostic marker supporting heightened vigilance, acknowledging conflicting predictive data across trials), high ctDNA burden (where validated assays are clinically available; see practical considerations below), and heavy overall disease burden. Patients with two or more of these features represent a biologically high-risk subgroup in whom combination therapy is most clinically plausible, though prospective validation of this stratification is not yet available.

Practical limitations of ctDNA in step 2 stratification. Several real-world barriers constrain the routine use of ctDNA as a treatment-selection variable outside clinical trials. Quantitative plasma profiling is not yet universally available; access is concentrated at academic centers and reference laboratories, with significant heterogeneity in assay sensitivity, panel breadth, allele-fraction reporting, and turnaround time (typically 1 to 3 weeks). Insurance and payer coverage for serial pretreatment ctDNA in EGFR-mutant NSCLC remains inconsistent in many jurisdictions outside the United States, and out-of-pocket costs can be prohibitive. Tumor shedding into the circulation varies widely with disease burden, anatomic distribution (lower shedding in CNS-only and indolent oligometastatic disease), and recent therapy, such that a low or undetectable ctDNA result does not reliably indicate low biological risk. No prospectively validated quantitative threshold currently defines a “high ctDNA” patient eligible for treatment intensification, and the cutoffs used in MARIPOSA and FLAURA2 exploratory analyses are not directly comparable across the assays used in routine practice. For all of these reasons, ctDNA should be applied in step 2 only where validated testing is available, only as one element of an integrated risk assessment alongside imaging and clinical features, and never as a sole criterion driving combination therapy selection. In settings where ctDNA is unavailable, the algorithm should be operationalized using the anatomic and clinical high-risk features (liver metastases, baseline CNS disease, heavy disease burden) and molecular co-alterations identified on tissue NGS, without loss of internal coherence.

Step 3: Patient fitness and comorbidity evaluation. Assess ECOG performance status, organ function (renal, hepatic, cardiac), VTE risk factors, frailty (using a validated tool such as the G8 or Clinical Frailty Scale), and polypharmacy burden. Patients with ECOG ≥ 2, significant organ dysfunction, frailty by validated assessment, or age ≥ 75 years with meaningful comorbidities should be directed toward single-agent osimertinib regardless of tumor biology.

Step 4: Patient preference, values, and logistical feasibility. Conduct a structured shared decision-making conversation. Patients who accept the higher toxicity burden associated with combination therapy, are motivated for intensive monitoring, and have reliable access to infusion or injection services are appropriate candidates. Those prioritizing quality of life, with access barriers, or declining the incremental risk of combination therapy should receive osimertinib monotherapy with transparent discussion of the benefit trade-off.

In patients meeting criteria for combination therapy, selection between FLAURA2 and MARIPOSA regimens should incorporate individual comorbidity profiles. FLAURA2 (osimertinib plus platinum-pemetrexed) may be preferred in patients with prior or active significant dermatologic conditions, a prior history of significant IRR, cardiac indications precluding anticoagulation required for MARIPOSA, or when a chemotherapy-containing approach is preferred for high-burden disease. MARIPOSA with SC amivantamab may be preferred when cytopenia risk is elevated, chemotherapy is otherwise contraindicated, or when a chemotherapy-free strategy is strongly preferred by a fit and informed patient.

**Table 2:** Algorithm summary (corresponding to Fig. 1)

Step	Criteria favoring combination therapy	Criteria favoring osimertinib monotherapy
1. Mutation Subtype	Ex19del or L858R (standard indication for both combination strategies)	Uncommon *EGFR* variants – manage per dedicated guidelines
2. Tumor Biology & Molecular Risk	Liver metastases; baseline CNS disease; *TP53* co-mutation (prognostic, not definitively predictive; conflicting data across trials); high ctDNA burden where validated testing is available; high disease burden; ≥2 high-risk features	Low metastatic burden; *TP53* wild-type; no visceral metastases; low/undetectable ctDNA where measured; limited disease extent
3. Patient Fitness	ECOG 0–1; adequate organ function; no significant comorbidity; age < 75 or biologically fit ≥ 75; acceptable polypharmacy; VTE prophylaxis feasible	ECOG ≥ 2; organ dysfunction; frailty (G8 or CFS); age ≥ 75 with comorbidities; high polypharmacy burden; anticoagulation contraindicated
4. Preference & Access	Accepts higher toxicity for potential OS gain; reliable SC injection or infusion access; motivated for monitoring; cost/coverage not a barrier	Prioritizes QoL or treatment convenience; limited access; declines incremental toxicity; significant cost or coverage barrier

CFS = Clinical Frailty Scale; CNS = central nervous system; ctDNA = circulating tumor DNA; ECOG = Eastern Cooperative Oncology Group; QoL = quality of life; SC = subcutaneous; VTE = venous thromboembolism.

If combination therapy is selected: prefer FLAURA2 (osimertinib + platinum–pemetrexed) when VTE prophylaxis/anticoagulation is contraindicated or dermatologic co-morbidity is a major concern. Prefer MARIPOSA (amivantamab + lazertinib, SC formulation preferred) when cytopenia risk is high, chemotherapy is contraindicated, or a chemotherapy-free approach is strongly preferred by the patient.

## Resistance mechanisms and future directions

Acquired resistance remains biologically inevitable across all currently available *EGFR*-directed strategies. The mechanisms of resistance following combination therapy differ meaningfully from those observed after osimertinib monotherapy, with downstream implications for post-progression treatment planning.

In MARIPOSA, resistance analyses demonstrated that upfront *EGFR*-MET dual blockade substantially suppresses the dominant osimertinib resistance mechanisms. *MET* amplification occurred in 13% of patients receiving osimertinib monotherapy but in only 3% receiving amivantamab-lazertinib (*P* = 0.002). Secondary *EGFR* mutations, particularly C797S, emerged in 8% of osimertinib patients compared with 1% in the combination arm (*P* = 0.01) [4, 5]. The diversity of resistance pathways was also narrower in the combination arm. For patients who do progress after amivantamab-lazertinib, the potential implications of a narrower, less heterogeneous resistance landscape are clinically meaningful but require prospective validation through ongoing post-progression studies.

Patients progressing after FLAURA2 may be salvaged with mechanism-directed agents targeting the resistance driver identified by liquid biopsy at progression, including *MET*-directed therapy for *MET* amplification (capmatinib, tepotinib) and emerging fourth-generation *EGFR* TKIs for tertiary *EGFR* resistance mutations. The MARIPOSA-2 trial established amivantamab plus chemotherapy as an effective post-osimertinib progression strategy, producing a median PFS of 6.3 months compared with 4.2 months for chemotherapy alone (HR 0.48; *P* < 0.001), providing a validated second-line option specifically for patients who received osimertinib monotherapy or FLAURA2 first-line [38]. Mandatory liquid biopsy at progression is strongly encouraged to guide mechanism-based salvage selection in all settings.

The most pressing scientific priority in this field is prospective biomarker-driven trial design. The phase 3 TOP trial (NCT06120140), reported at ELCC 2026, provides the first prospective evidence that patients with *EGFR*-*TP53* co-mutations derive a doubling of PFS from osimertinib plus carboplatin-pemetrexed compared with monotherapy [24]. Beyond *TP53*, systematic prospective evaluation of *CDKN2A*, *RB1*, *MET* copy number, and quantitative ctDNA burden as selection variables is needed. Biomarker-adaptive platform trials in which randomization is conditioned on prospectively defined molecular profiles represent the most efficient design to address questions that current exploratory analyses cannot definitively answer.

Real-world evidence registries will be indispensable for contextualizing trial findings in broader populations. FLAURA2 and MARIPOSA enrolled relatively young, fit, predominantly Asian populations with ECOG 0–1. Population-level data from patients with comorbidities, elderly patients, and those receiving combination therapy in community settings will be necessary to understand the absolute magnitude and durability of benefit outside controlled trial conditions.

### Validation cohorts for the proposed algorithm

The proposed algorithm has not been prospectively validated, and several complementary cohorts are well positioned to provide that validation. The MARIPOSA and FLAURA2 biomarker subsets, including the MARIPOSA secondary high-risk analysis [18] and the FLAURA2 plasma EGFR-mutant subset, can be re-analyzed using the integrated step-2 risk categories to test whether algorithm-classified high-risk patients show the largest absolute PFS and OS gains; both sponsors have released individual-participant-level biomarker datasets through controlled access, enabling external academic validation. The phase 3 TOP cohort [24] offers a directly relevant prospective validation set for the *TP53*-co-mutated subgroup, and the ongoing COPERNICUS pragmatic study [34] will contribute data from a clinically representative US and EMEA population that includes patients with ECOG 2, organ dysfunction, and prior thrombotic events not eligible for the registrational trials. Population-scale evidence is increasingly accessible through Flatiron Health-Foundation Medicine linked clinico-genomic databases (CGDB), the AACR Project GENIE Biopharma Collaborative (GENIE BPC) NSCLC cohort, and the ctMoniTR longitudinal ctDNA repository; these datasets contain thousands of treated *EGFR*-mutant patients with linked molecular profiling, treatment exposure, and survival outcomes, and can support retrospective external validation of the algorithm with adjustment for measurable confounders. The definitive test, however, will require a dedicated prospective biomarker-stratified study, ideally a multi-arm platform trial that prospectively classifies patients into high-risk and standard-risk strata at diagnosis and randomizes within each stratum to combination versus single-agent therapy, with PFS, OS, patient-reported outcomes, and serial ctDNA kinetics as co-primary and key secondary endpoints. Such a trial would directly establish whether the algorithm-defined high-risk classification predicts differential benefit, and would close the present gap between exploratory subgroup signals and practice-changing evidence.

### Impact of co-occurring molecular alterations on algorithm recommendations

Co-occurring molecular alterations meaningfully modulate the prognostic and possibly the predictive interpretation of step 2, and a more granular reading is warranted than is captured by a single *TP53* binary. Domain-specific *TP53* mutations, particularly within the DNA-binding domain and especially in exon 5, appear to confer substantially worse osimertinib outcomes than mutations in other domains, suggesting that an algorithm-driven recommendation for intensification may be more justified in DNA-binding-domain mutants than in *TP53*-co-mutated patients overall [25]. Co-alterations in *CDKN2A* and *RB1*, when present concurrently with *TP53*, identify a compound-suppressor phenotype with the worst osimertinib outcomes in retrospective series and elevated risk of histologic transformation; in these patients, the algorithm should weight toward combination therapy even in the absence of dominant anatomic high-risk features [27]. *PIK3CA* co-alteration, in a US multi-institutional cohort, was associated with significantly worse OS only when paired with *TP53*, reinforcing the need to interpret tumor suppressor co-alterations in combination rather than in isolation [26]. Baseline *MET* copy number gain, although uncommon at diagnosis, biologically supports preferential use of an EGFR-MET bispecific approach (MARIPOSA) over a chemotherapy-based combination (FLAURA2), since amivantamab directly targets MET; this is a reasonable consideration when both regimens are otherwise on the table. Practically, these refinements require a comprehensive tissue-based NGS panel rather than a hotspot *EGFR* assay, and where such testing is unavailable, the algorithm should default to clinical and anatomic risk features without overstating molecular precision.

### Translating the algorithm into real-world clinical practice

Translating a risk-adapted framework into routine practice depends as much on system-level readiness as on individual clinical judgment. Comprehensive tissue NGS turnaround commonly exceeds two weeks, particularly outside academic centers, and treatment cannot be deferred indefinitely; pragmatic workflows should combine a rapid hotspot *EGFR* result to permit timely osimertinib initiation with a reflex comprehensive NGS panel whose results inform the formal step-2 reassessment and decision to escalate or maintain monotherapy. Payer and insurance heterogeneity, prior-authorization timelines, and out-of-pocket cost, particularly for amivantamab-based regimens whose annual cost exceeds two hundred thousand US dollars in many systems, are concrete barriers; embedding financial navigation early in the workflow rather than at the point of prescription writing materially improves adherence. Infusion or injection capacity is unevenly distributed; the availability of SC amivantamab with administration time under five minutes substantially eases this constraint, but does not eliminate it in rural and community practices where weekly to biweekly visits remain logistically difficult. Frailty assessment uptake is low outside dedicated geriatric oncology services, yet most patients with *EGFR*-mutant NSCLC in real-world practice are older than the trial median age; institutional pathways should include a brief validated screen (G8 or Clinical Frailty Scale) at the time of first oncology consult, with a low threshold for a formal geriatric oncology evaluation in patients flagged as at risk. Geographic disparities in molecular testing access disproportionately affect rural, underinsured, and minority populations and risk widening, rather than narrowing, existing outcome gaps; equity-conscious implementation should pair the algorithm with telemedicine-enabled molecular tumor board access and reimbursement advocacy. Concrete implementation levers include institutional clinical pathways that embed the four algorithm steps into the electronic health record at the point of treatment ordering, integration with the NCCN Clinical Pathways platform where used, decision-support tools that surface high-risk feature combinations to prescribing oncologists, structured geriatric oncology consult workflows for patients over seventy-five years or with frailty signals, and standing prophylaxis order sets for VTE and dermatologic toxicity when amivantamab-lazertinib is selected. With these system-level supports in place, the algorithm becomes a practical tool rather than an aspirational framework.

### Limitations

Several limitations of the present synthesis warrant explicit acknowledgment. First, the proposed algorithm has not been prospectively validated; its construct rests on landmark trial evidence, exploratory biomarker subgroup analyses, and retrospective cohort data, none of which constitute level-1 evidence for treatment-selection. Second, the central biomarker arguments for step 2, particularly *TP53* co-mutation and baseline ctDNA, derive from unplanned post hoc subgroup analyses (MARIPOSA, FLAURA2) that were not powered for formal interaction testing and have produced inconsistent predictive directionality across trials; the TOP results in *TP53*-co-mutated patients are an important first prospective signal but remain immature for OS. Third, quantitative ctDNA profiling is not universally available and lacks a prospectively validated treatment-selection threshold, constraining one of the algorithm’s stratification variables in real-world practice. Fourth, the evidence base is evolving rapidly: COPERNICUS, the FLAURA2 long-term resistance program, post-progression studies after MARIPOSA-based first-line therapy, and ongoing fourth-generation EGFR TKI and ADC trials will materially update the considerations described here. Fifth, the registrational trial populations underlying our framework are skewed toward ECOG 0–1 patients with median ages in the early to mid sixties and, for MARIPOSA, a substantial proportion of Asian participants; extrapolation to older, frailer, comorbid, and non-Asian populations carries uncertainty. Sixth, this review is a narrative synthesis rather than a systematic or PRISMA-compliant review; literature was selected on the basis of clinical relevance and topical fit, and a formal risk-of-bias assessment of included studies was not performed. Seventh, resistance pathway data are early and based predominantly on plasma ctDNA samples with limited tissue confirmation, particularly for histologic transformation, and the post-MARIPOSA salvage landscape remains underdeveloped relative to the post-osimertinib setting. Eighth, treatment cost and payer coverage vary substantially by jurisdiction; recommendations that are practical in one health system may be infeasible in another. These limitations do not invalidate the value of a structured risk-adapted framework, but they delimit its current evidentiary footing and identify the prospective work required before any element of the algorithm can be regarded as practice-defining.

## Conclusion

The concurrent availability of three evidence-supported first-line options for *EGFR*-mutant advanced NSCLC marks a genuine inflection point in the field. Combination therapy with amivantamab-lazertinib (MARIPOSA) or osimertinib plus platinum-pemetrexed (FLAURA2) delivers statistically significant and clinically meaningful OS benefits over monotherapy. These are real and important advances. The phase 3 TOP trial, reported in 2026, provides the first prospective evidence that a molecularly defined subgroup (concurrent *EGFR* and *TP53* mutations) derives a doubling of PFS from osimertinib plus chemotherapy; this is the strongest contemporary signal that biomarker-driven intensification is feasible, although mature OS data are awaited [24].

Yet these survival benefits arrive with substantially greater toxicity, final analysis grade ≥ 3 event rates of 70% and 80%, respectively that are not equally acceptable, accessible, or safe across the full clinical spectrum of patients with *EGFR*-mutant NSCLC. The risk-adapted framework proposed here integrates tumor biology, molecular markers, patient fitness, treatment preference, and logistical realities into a clinically operable structure. It argues for deliberate, criteria-based selection of combination therapy rather than its universal application. High-risk anatomic features, particularly liver metastases and baseline CNS disease, and *TP53* co-mutation as a prognostic signal provide the strongest current rationale for intensification, while acknowledging that the predictive value of molecular markers remains unvalidated prospectively. Elderly, frail, or preference-discordant patients retain a fully appropriate and evidence-supported pathway to single-agent osimertinib.

Prospective validation of treatment-selection algorithms in biomarker-enriched trials is the field’s highest scientific priority. Until that evidence matures, clinical judgment grounded in the evidence synthesized here, combined with honest, structured conversations with patients about benefit and burden, represents the most defensible approach to navigating this expanding landscape.

## Data Availability

No datasets were generated or analysed during the current study.

## Abbreviations

ASCO: American Society of Clinical Oncology
CI: Confidence Interval
CNS: Central Nervous System
ctDNA: Circulating Tumor DNA
ECOG: Eastern Cooperative Oncology Group
FDA: Food and Drug Administration
HR: Hazard Ratio
IRR: Infusion-Related Reactions
IV: Intravenous
NCCN: National Comprehensive Cancer Network
NSCLC: Non-Small Cell Lung Cancer
OS: Overall Survival
PFS: Progression-Free Survival
SC: Subcutaneous
TKI: Tyrosine Kinase Inhibitor
VTE: Venous Thromboembolic Events

## Declarations

### Compliance with reporting guidelines

This narrative review adheres to the general principles of good narrative review practice. REMARK criteria for prognostic biomarker reporting have been referenced where applicable [10]. Transparent reporting of evidence quality, study limitations, and evidence grading has been incorporated throughout the manuscript.
